# Clusters of home- and community- based service use and association with quality of life in older adults

**DOI:** 10.1093/geroni/igaf122.2824

**Published:** 2025-12-31

**Authors:** Eric Jutkowitz, Romil Parikh, Jack Wolf, Yinfei Duan, Benjamin Langworthy, Tetyana Shippee

**Affiliations:** University of Minnesota, Minneapolis, Minnesota, United States; University of Minnesota School of Public Health, Minneapolis, Minnesota, United States; University of Minnesota School of Public Health, Minneapolis, Minnesota, United States; University of Alberta, Edmonton, Alberta, Canada; University of Minnesota School of Public Health, Minneapolis, Minnesota, United States; University of Minnesota, Minneapolis, Minnesota, United States

## Abstract

Measuring the quality of home- and community-based services (HCBS) is challenging because people use a diverse array of services. To fill this gap we evaluated whether distinct clusters of older adults exist based on their patterns of HCBS use, and whether consumer-reported quality of life (QoL) varies between these clusters, with or without dementia. We included 775 Medicaid HCBS consumers in Minnesota who also participated in the 2017-2018 National Core Indicators-Aging & Disability Adult ConsumerSurvey (NCI-AD). We linked NCI-AD respondents with their Medicaid claims. A validated QoL score was constructed using factor analysis from items in the NCI-AD. The QoL score includes items reflecting care experiences, security, and autonomy. Principal component analysis identified clusters of consumers with similar patterns of HCBS use (home healthcare, non-medical transportation, personal care assistance, adult day care, durable medical equipment, and homemaker services). We evaluated differences in mean QoL scores between clusters and within each cluster among consumers with and without dementia, using linear regression. Four clusters were identified based on patterns in service type. The clusters varied in composition based on race/ethnicity, proportion with dementia, and functional dependence. In regression analyses, mean QoL scores were similar between clusters. Within each cluster, mean QoL scores were also similar between those with and without dementia. These findings indicate that despite significant heterogeneity in patterns of HCBS use, QoL scores can be used reliably as a standard quality indicator for all HCBS consumers, regardless of dementia diagnosis, to inform HCBS quality assurance efforts.

